# Online Patient Education in Obstructive Sleep Apnea: ChatGPT versus Google Search

**DOI:** 10.3390/healthcare12171781

**Published:** 2024-09-05

**Authors:** Serena Incerti Parenti, Maria Lavinia Bartolucci, Elena Biondi, Alessandro Maglioni, Giulia Corazza, Antonio Gracco, Giulio Alessandri-Bonetti

**Affiliations:** 1 Unit of Orthodontics and Sleep Dentistry, Department of Biomedical and Neuromotor Sciences (DIBINEM), University of Bologna, Via San Vitale 59, 40125 Bologna, Italy; serena.incerti@unibo.it (S.I.P.); maria.bartolucci3@unibo.it (M.L.B.); alessandro.maglioni@studio.unibo.it (A.M.); g4corazza@gmail.com (G.C.); giulio.alessandri@unibo.it (G.A.-B.); 2 Postgraduate School of Orthodontics, University of Bologna, Via San Vitale 59, 40125 Bologna, Italy; 3 Postgraduate School of Orthodontics, Department of Neurosciences, Section of Dentistry, University of Padua, 35122 Padua, Italy; antonio.gracco@unipd.it

**Keywords:** obstructive sleep apnea, patient education, artificial intelligence, search engine

## Abstract

The widespread implementation of artificial intelligence technologies provides an appealing alternative to traditional search engines for online patient healthcare education. This study assessed ChatGPT-3.5’s capabilities as a source of obstructive sleep apnea (OSA) information, using Google Search as a comparison. Ten frequently searched questions related to OSA were entered into Google Search and ChatGPT-3.5. The responses were assessed by two independent researchers using the Global Quality Score (GQS), Patient Education Materials Assessment Tool (PEMAT), DISCERN instrument, CLEAR tool, and readability scores (Flesch Reading Ease and Flesch–Kincaid Grade Level). ChatGPT-3.5 significantly outperformed Google Search in terms of GQS (5.00 vs. 2.50, *p* < 0.0001), DISCERN reliability (35.00 vs. 29.50, *p* = 0.001), and quality (11.50 vs. 7.00, *p* = 0.02). The CLEAR tool scores indicated that ChatGPT-3.5 provided excellent content (25.00 vs. 15.50, *p* < 0.001). PEMAT scores showed higher understandability (60–91% vs. 44–80%) and actionability for ChatGPT-3.5 (0–40% vs. 0%). Readability analysis revealed that Google Search responses were easier to read (FRE: 56.05 vs. 22.00; FKGL: 9.00 vs. 14.00, *p* < 0.0001). ChatGPT-3.5 delivers higher quality and more comprehensive OSA information compared to Google Search, although its responses are less readable. This suggests that while ChatGPT-3.5 can be a valuable tool for patient education, efforts to improve readability are necessary to ensure accessibility and utility for all patients. Healthcare providers should be aware of the strengths and weaknesses of various healthcare information resources and emphasize the importance of critically evaluating online health information, advising patients on its reliability and relevance.

## 1. Introduction

Obstructive sleep apnea (OSA) affects approximately 1 billion people worldwide between the ages of 30 and 69, and is likely to become an increasingly significant global issue as obesity rates rise and the population continues to age [1]. Despite being recognized as a potentially life-threatening chronic condition with significant economic and social burdens, OSA remains underdiagnosed and undertreated [1,2]. Moreover, awareness and knowledge of OSA among the general population are currently poor [3]. The patient’s self-report of risk factors and symptoms can be critical for the early detection of OSA. Therefore, appropriate online patient education on OSA-related information can facilitate early diagnosis through increased disease awareness and promote adherence to prescribed management.

Patients are increasingly seeking health information online [4,5,6,7]. This is challenging due to the information overload on the web, the variability in source reliability, the varying levels of health literacy required to comprehend some content, and the expertise needed to accurately locate the correct information [8]. Notably, most online health resources regarding OSA may not always be reliable or easily understandable, often providing insufficient content that fails to address the most important aspects of the disease [9,10,11,12].

The rapid and widespread implementation of generative artificial intelligence (AI) using large language models to interpret language and produce new content, simulating human-like responses to user inquiries, offers a more appealing alternative to traditional web search engines [13]. This innovation is particularly relevant to people living with chronic conditions, who more frequently rely on online resources to manage their health. In this context, Chat Generative Pre-trained Transformer (ChatGPT, OpenAI, San Francisco, CA, USA), introduced in late 2022, has an estimated over 180 million users with approximately 600 million visits per month. Given its rapidly growing popularity and promising performance as a source of healthcare knowledge [14,15], ChatGPT could potentially replace traditional search engines such as Google Search (the most used search engine in the world, with 158 million visits per month; Mountain View, CA, USA) as a primary source for patient inquiries regarding OSA.

A recent systematic review on the utility of ChatGPT in healthcare education highlights the need for further studies to determine the content of language models and their potential impact in healthcare settings due to the risk of generating inaccurate content, including hallucinations [16]. In the context of AI, a hallucination refers to a confident response generated by an AI model that lacks justification based on its training data and, consequently, is not grounded in reality or empirical evidence [16]. Consequently, there is concern about the implications if patients shift from “Dr. Google” to “Dr. ChatGPT”, given the possible inaccuracies in the generated responses. Previous studies have evaluated the quality and readability of AI chatbot responses on OSA, but the completeness and trustworthiness of the information provided have not yet been thoroughly assessed [17,18,19]. Additionally, no comparisons have been made between the chatbot’s responses and traditional web search engines commonly used in online patient education. The aim of this paper is to critically analyze the quality, reliability, and readability of ChatGPT’s answers to the most common patient queries on OSA, using Google Search as a benchmark, to provide a more comprehensive assessment of the chatbot’s utility and efficacy. A novel contribution of the present study is the evaluation of the completeness, accuracy, evidence-based nature, and relevance of the provided information.

## 2. Materials and Methods

The term “obstructive sleep apnea” was entered into Google Search in incognito mode on 15 December 2023. The top 10 most searched questions (Table 1), along with their respective answers and references provided by Google Search, were recorded. These same 10 questions were then entered into ChatGPT-3.5, followed by the prompt “provide references”. The resulting answers and references were extracted and anonymized.

The answers were evaluated for quality, reliability, and readability by two independent researchers with a master’s degree in sleep dentistry who have been working at the Orthodontics and Sleep Dentistry unit of the University of Bologna, Italy, for more than ten years (S.I.P. and M.L.B.). These researchers were blinded to the source of the information. Any discrepancies were resolved through discussion with a third researcher (E.B.) until consensus was reached. The reference links provided by both Google Search and ChatGPT-3.5 were accessed and recorded.

### 2.1. Quality and Reliability Assessment

The answers were evaluated using the Global Quality Score (GQS) [20] and the Patient Education Materials Assessment Tool (PEMAT) [21,22], both of which are validated instruments for assessing information quality. The GQS, a widely used tool, utilizes a 5-point Likert scale ranging from 1 (poor quality, most information missing, not beneficial for patients) to 5 (excellent quality and information flow, very useful for patients) to measure overall information quality. The PEMAT, developed by the Agency for Healthcare Research and Quality, includes PEMAT-P for printable materials and consists of 26 items that yield two percentage scores: one for understandability (i.e., consumers of diverse backgrounds and varying levels of health literacy can process and explain key message) and one for actionability (i.e., consumers of diverse backgrounds and varying levels of health literacy can identify what they can do based on the information presented).

For each item, except those marked as not applicable (N/A), a score of 1 point (agree) is given when a characteristic is consistently present throughout the material, and 0 points (disagree) when there are clear instances where the characteristic could have been better addressed. The percentage scores for understandability and actionability are calculated by excluding the items scored as N/A. Higher scores indicate a superior quality of information.

To evaluate reliability, the DISCERN instrument was employed [23]. Developed by a specialized team at the University of Oxford, United Kingdom, to assess the quality of written consumer health information, the DISCERN tool comprises 16 items, each scored on a scale from 1 to 5. The total score ranges from 16 to 80 points. The 16 items are divided into three sections: section 1 (items 1–8) evaluates the publication’s reliability, section 2 (items 9–15) assesses the quality of the information, and section 3 (item 16) provides an overall quality rating. A higher DISCERN score indicates greater information reliability and a more balanced presentation of content.

### 2.2. Content Evaluation

The CLEAR tool was utilized to specifically assess the quality of health-related content [24]. This instrument comprises five items: (1) completeness, (2) lack of false information, (3) evidence, (4) appropriateness, and (5) relevance. Each item is rated on a 5-point Likert scale, ranging from poor to excellent. For descriptive interpretation, the CLEAR item scores are classified into the following categories: scores of 1–1.79 are deemed “poor”, 1.80–2.59 “satisfactory”, 2.60–3.39 “good”, 3.40–4.19 “very good”, and 4.20–5.00 “excellent”. The total CLEAR score ranges from 5 to 25 and is divided into three categories: 5–11 indicates “poor” content, 12–18 “average” content, and 19–25 “very good” content.

### 2.3. Readability Assessment

Readability was assessed using the Flesch Reading Ease (FRE) score, the Flesch–Kincaid Grade Level (FKGL) score, and the word count for each answer [25,26]. The FRE score ranges from 0 to 100 and indicates the ease with which a text can be understood, with lower scores corresponding to higher difficulty reading levels. The FKGL score evaluates the educational level required to comprehend the text and its complexity, with higher values indicating more difficult content.

### 2.4. Statistical Analysis

The normality of the data was evaluated utilizing the Kolmogorov–Smirnov test. To compare the scores of GQS, DISCERN, CLEAR, and readability between ChatGPT-3.5 and Google Search, the Mann–Whitney U test was applied. The chi-square test was employed for analyzing PEMAT-P scores. All statistical analyses were performed using SPSS for Windows (version 18.0; 2009; SPSS Inc., Chicago, IL, USA). Statistical significance was determined at a threshold of *p* < 0.05.

## 3. Results

### 3.1. Quality and Reliability Assessment 

**Figure 1:**
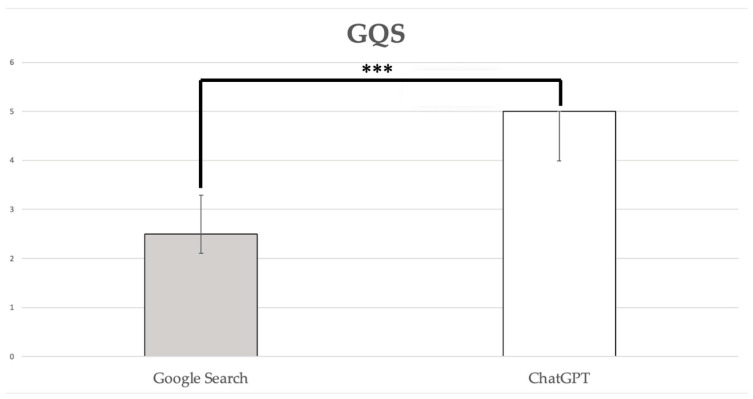
GQS scores. Data are reported as medians with a 95% confidence interval: ***, *p* < 0.0001.

The median GQS for answers provided by Google Search was 2.50, with a 95% confidence interval (CI) of 2.11–3.29, indicating generally poor quality, with some information present but of very limited use to patients. In contrast, the median GQS for answers provided by ChatGPT-3.5 was 5.00 (3.99–5.00), denoting high-quality content that is useful to patients and covers most relevant information. This difference between Google Search and ChatGPT-3.5 was statistically significant (*p* < 0.0001; Figure 1).

**Figure 2:**
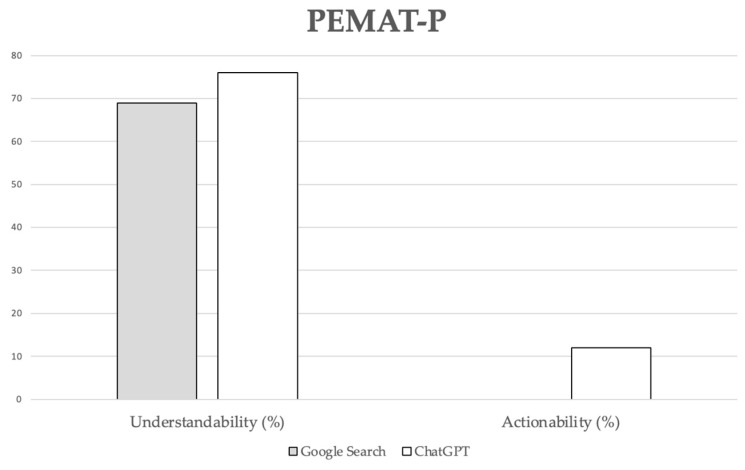
PEMAT-P scores. Data are reported as mean percentages. No statistically significant differences were observed between the groups for these variables (*p* = 0.067 for understandability; *p* = 0.060 for actionability).

PEMAT-P understandability scores ranged from 44% to 80% for Google Search and from 60% to 91% for ChatGPT-3.5. Google Search consistently scored 0% for PEMAT-P actionability, while ChatGPT-3.5 scores ranged from 0% to 40%. Despite a trend toward producing more comprehensible and actionable content by ChatGPT-3.5, no statistically significant differences were observed between the groups for these variables (Figure 2).

**Figure 3:**
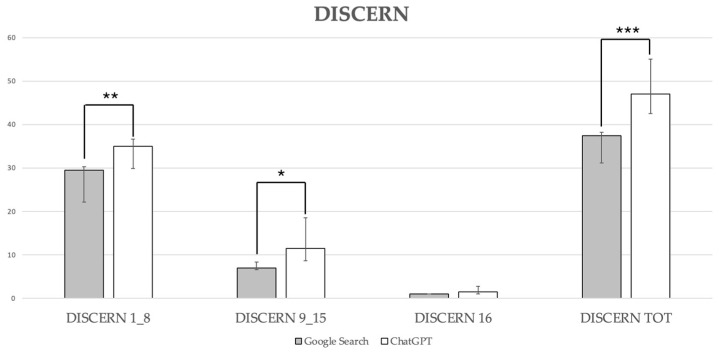
DISCERN scores. Data are reported as medians with a 95% confidence interval: DISCERN 1_8, **, *p* = 0.001; DISCERN 9_15, *, *p* = 0.02, DISCERN total, ***, *p* < 0.0001.

Google Search scored significantly lower compared with ChatGPT-3.5 for reliability (DISCERN 1_8; Google Search: 29.50 (22.13–30.27) versus ChatGPT-3.5: 35.00 (29.89–36.71), *p* = 0.001), quality of information (DISCERN 9_15; Google Search: 7.00 (6.60–8.41) versus ChatGPT-3.5: 11.50 (8.62–18.58), *p* = 0.02), while no statistically significant difference was detected for DISCERN 16 (Google Search consistently scored 1.00 versus ChatGPT-3.5: 1.50 (1.04–2.76), *p* = 0.063). The median DISCERN total score for answers provided by Google Search was 37.50, with a 95% CI of 31.15–38.25, indicating generally poor quality. In contrast, the median DISCERN total score for answers provided by ChatGPT-3.5 was 47.00 (42.51–55.10), denoting an overall fair quality. This difference between Google Search and ChatGPT-3.5 was statistically significant (*p* < 0.0001; Figure 3).

### 3.2. Content Evaluation

**Figure 4:**
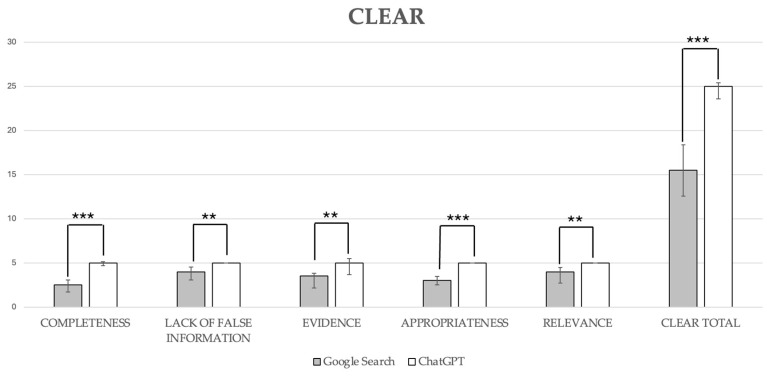
CLEAR scores. Data are reported as medians with a 95% confidence interval. Completeness, ***, *p* < 0.0001; lack of false information, **, *p* = 0.002; evidence, **, *p* = 0.001; appropriateness, ***, *p* < 0.0001; relevance, **, *p* = 0.002; CLEAR total score, ***, *p* < 0.0001.

Google Search scored significantly lower compared with ChatGPT-3.5 across all CLEAR metrics, with Google Search results ranging from satisfactory to very good, while ChatGPT-3.5 consistently achieved excellent scores. Overall, Google Search fared worse than ChatGPT-3.5 in CLEAR total scores, with median scores of 15.50 (12.59–18.40) for Google Search compared to 25.00 (23.59–25.41) for ChatGPT-3.5, a difference that was statistically significant (*p* < 0.001; Figure 4). ChatGPT provided sources linked to scientific articles that were readily accessible for 9 out of 10 questions.

### 3.3. Readability Assessment 

**Figure 5:**
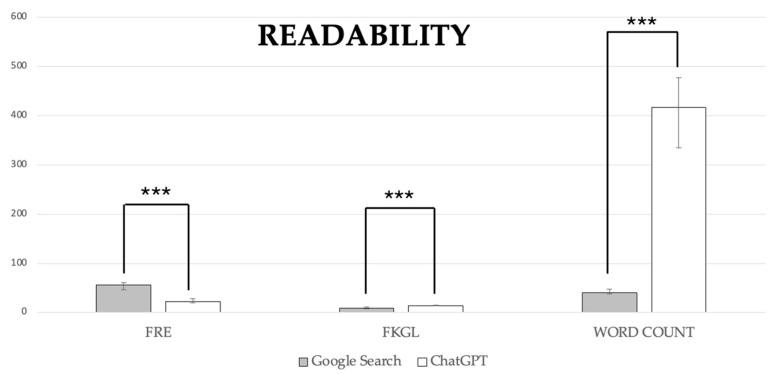
Readability scores. Data are reported as medians with a 95% confidence interval. ***, *p* < 0.0001 for all the examined variables.

The answers provided by Google Search were less difficult to read compared with those from ChatGPT-3.5 (*p* < 0.0001 for all the examined variables; Figure 5). A statistically significant difference between the groups was found for FRE scores, with Google Search scoring 56.05 (46.32–60.48) versus ChatGPT-3.5 at 22.00 (20.10–28.74). For FKGL, Google Search had a score of 9.00 (7.76–11.09) compared to ChatGPT-3.5’s 14.00 (13.31–14.93). Additionally, word count showed a significant difference, with Google Search averaging 41.00 (38.01–47.19) words versus ChatGPT-3.5’s 417 (335.07–477.53).

## 4. Discussion

This study was the first to carry out a systematic comparative analysis of ChatGPT-3.5’s responses to the 10 most frequently queried questions related to OSA against a traditionally used resource of online patient education, Google Search. The principal aspects of OSA were comprehensively addressed, covering the disease’s definition, primary symptoms, significance, management strategies, etiology, prognosis, and recommended lifestyle modifications. This was achieved through the analysis of the top 10 most searched questions. ChatGPT-3.5 was used with minimal prompting to better simulate realistic usage by patients. Previous studies have evaluated ChatGPT-3.5’s accuracy in providing information related to OSA [17,18,19]; however, they have not sufficiently addressed the completeness, appropriateness, evidence-based nature, and relevance of the information in responding to patient inquiries.

ChatGPT-3.5 outperformed Google Search in terms of quality, as evidenced by significantly higher median scores on the GQS and DISCERN. The GQS scores suggest that while Google Search provides information, it tends to be of poor quality and limited usefulness to patients. Conversely, the responses provided by ChatGPT-3.5 demonstrate high quality, comprehensively addressing the key topics within each analyzed dimension. The statistically significant difference in scores between the two resources indicates that patients may find ChatGPT-3.5 more informative and useful compared to Google Search when seeking healthcare information related to OSA.

In accordance with these findings, data from the PEMAT scores demonstrated a tendency for the information generated by ChatGPT-3.5 to be more comprehensible to consumers from diverse backgrounds and varying levels of health literacy, successfully guiding patients on how to address their health concerns. However, it is important to note that there were no statistically significant differences observed between the groups for these variables.

The CLEAR instrument provided more in-depth insights into the responses generated by the resources. It yielded statistically significant higher scores for ChatGPT-3.5, which regularly ranked in the “excellent” range across metrics of completeness, accuracy (including the absence of false information), evidence-based content, and topical appropriateness and relevance (clear, concise, and easy to understand content, free from irrelevant information). In contrast, the total CLEAR score for Google Search responses was categorized as “average”.

The most significant difference between the two analyzed resources was found in the completeness of health information. Evaluating completeness—defined as the optimal provision of information, avoiding excess or insufficiency, and identifying potential gaps—is crucial. Comprehensive health information empowers laypeople to make informed health decisions and improves communication with healthcare professionals. Insufficient information can result in incorrect self-diagnosis and the associated risk of delayed seeking of medical consultation [24]. Notably, Google Search scored significantly lower compared to ChatGPT-3.5, exhibiting major deficiencies in areas such as definition, etiology, disease significance, and recommended lifestyle modifications.

Additionally, Google Search underperformed relative to ChatGPT-3.5 in terms of accuracy and evidence-based content. Evaluating potential false content in health information is critical for mitigating the risk of incorrect self-diagnosis, delayed seeking of medical consultation, and undermining trust in healthcare professionals. Google Search exhibited significant deficiencies in domains related to prognosis. An essential aspect of health information provision is ensuring it is supported by the latest scientific advances and free from bias or misinformation. The key areas affected included management strategies, prognosis, and recommended lifestyle modifications. Notably, ChatGPT provided sources linked to scientific articles that were readily accessible for 9 out of 10 questions.

The information provided by Google Search was frequently unclear and ambiguous and often included irrelevant topics unrelated to the health query. This can hinder laypeople’s ability to discern information applicable to their health situation. The key areas that were most affected included the definition, prognosis, and significance of the disease. 

Google Search provided answers with higher readability scores (as measured by FRE and FKGL) compared to ChatGPT-3.5. This implies that Google’s responses are written in simpler language and are more accessible to the average adult. The significantly higher word count in answers generated by ChatGPT-3.5 suggests that its responses are more detailed and comprehensive, which could potentially overwhelm users seeking quick and concise information.

Consistent with the findings of this study, previous research has demonstrated that most online health resources regarding OSA are unreliable and inaccurate. The majority of websites provide insufficient and biased content, potentially increasing the likelihood of misinformation [9,10,11], while recent studies investigating ChatGPT have generally found that it provides appropriate answers to most questions on OSA patient education, without including incorrect or dangerous information in its responses [17,18,19].

Patients may find ChatGPT-3.5 more reliable and informative due to its higher quality scores and comprehensive content, despite its lower readability scores compared to Google Search. While Google Search provides information in simpler language, its content may lack completeness, appropriateness, accuracy, evidence-based nature, and relevance compared to ChatGPT-3.5’s responses, particularly regarding prognosis and disease significance. Promoting accurate information about OSA and its main aspects is crucial for addressing this potentially life-threatening disease and may also help reduce OSA underdiagnosis and undertreatment. Users seeking quick, easily digestible information may prefer Google Search, whereas those looking for more comprehensive information may benefit from ChatGPT-3.5’s responses. Healthcare providers should be aware of the strengths and weaknesses of various healthcare information resources and emphasize the importance of critically evaluating online health information, advising patients on its reliability and relevance.

A recent guide by the World Health Organization aims to equip policymakers and health professionals to provide better access to effective therapeutic patient education for all patients living with chronic conditions [27]. Notably, it underscores the importance of health professionals being skilled in assessing the quality of online information and supporting patients in navigating and evaluating what is likely to be useful and evidence-based. This aligns with the concept that lower health literacy—defined as the degree to which individuals can obtain, process, and understand basic health information needed to make appropriate health-related decisions—is associated with poorer outcomes, including lower health knowledge and an increased incidence of chronic illness [28,29].

ChatGPT-3.5 is undoubtedly a powerful tool, but some limitations must be acknowledged, and its responses should be interpreted with caution [30,31,32,33,34]. First, it may lack the ability to interpret complex medical nuances and might not always align with the latest clinical guidelines. Second, ChatGPT-3.5’s responses can vary based on the quality of its training data, potentially leading to inaccuracies or omissions. As previously mentioned, the tool often hallucinates, a challenge that technology creators are currently addressing. Third, slight adjustments in the wording or tone of a question can yield different answers [17], which can be misleading for patients seeking health-related information. Notably, the reliability and usefulness of AI-generated data can vary significantly based on the complexity and nuance of the inquiry, especially when assessed by experts in the field. If language models like ChatGPT-3.5 can be further investigated and improved, these tools have the potential to become valuable partners in patient care.

This study is limited by its focus on commonly asked patient questions formulated using a single type of wording. While this approach was intended to simulate realistic patient inquiries, we recognize that it may not capture the complexity of more detailed or specific medical questions that are better addressed through direct consultation with healthcare professionals. Additionally, slight changes in the wording or tone of questions can lead to different responses, particularly with AI models like ChatGPT-3.5. Future research should aim to investigate a broader spectrum of questions, particularly those requiring more nuanced and detailed responses, and should also assess the impact of variations in question phrasing on the outcomes. The quality of the training data could also have an impact on the consistency and reliability of the information provided by ChatGPT-3.5. To address this limitation in future research, it would be beneficial to conduct systematic evaluations of how variations in training data quality affect the performance of the outcomes. This could involve comparing responses generated using diverse datasets, including those with higher quality and more specialized content, to assess consistency and reliability. It is also important to acknowledge that the observed differences between ChatGPT and Google Search are specific to OSA and may not necessarily extend to other diseases, particularly those with a more extensive medical history.

## 5. Conclusions

AI technologies like ChatGPT can play a crucial role in disseminating health information that is both accurate and accessible, potentially reducing disease underdiagnosis and undertreatment. This can enhance patient knowledge and engagement, particularly concerning highly prevalent, potentially life-threatening, and chronic diseases such as OSA. The results of the present study underscore ChatGPT-3.5’s potential in improving the quality and comprehensiveness of online health information compared to Google Search, especially regarding prognosis and disease significance. However, they also highlight the importance of considering the readability of this information to ensure it is accessible and useful to all patients. Future AI research and development should address these challenges, potentially by integrating user feedback or adopting plain language guidelines to enhance accessibility. The applicability of AI tools across various healthcare domains warrants further exploration. Future studies should also examine how slight changes in the wording or tone of questions impact the reliability and utility of AI-generated data.

## Figures and Tables

**Table 1:** The top 10 most searched questions on obstructive sleep apnea.

n.	Questions
1	What are 3 symptoms of obstructive sleep apnea?
2	What is obstructive sleep apnea syndrome?
3	What’s the difference between sleep apnea and obstructive sleep apnea syndrome?
4	Is obstructive sleep apnea a serious illness?
5	Can obstructive sleep apnea be cured?
6	What is the main cause of sleep apnea?
7	What is the life expectancy of someone with sleep apnea?
8	Does sleep apnea go away?
9	What is the best position to sleep in with sleep apnea?
10	How can I prevent sleep apnea naturally?

## Data Availability

Dataset is available on request from the authors.
